# Ward-Based Non-Invasive Ventilation in Acute Exacerbations of COPD: A Narrative Review of Current Practice and Outcomes in the UK

**DOI:** 10.3390/healthcare6040145

**Published:** 2018-12-09

**Authors:** Samuel P. Trethewey, Ross G. Edgar, Alice M. Turner, Rahul Mukherjee

**Affiliations:** 1 Respiratory Medicine & Physiology, Birmingham Heartlands Hospital, University Hospitals Birmingham NHS Foundation Trust, Birmingham B95SS, UK; s-trethewey@doctors.org.uk (S.P.T.); a.m.turner@bham.ac.uk (A.M.T.); 2 Therapy Services, University Hospitals Birmingham NHS Foundation Trust, Birmingham B152TH, UK; r.edgar@bham.ac.uk; 3 Institute of Applied Health Research, University of Birmingham, Birmingham B152TT, UK; 4 Institute of Clinical Sciences, University of Birmingham, Birmingham B152TT, UK

**Keywords:** Chronic obstructive pulmonary disease, COPD, acute exacerbation, non-invasive ventilation, NIV, ward-based, do not intubate, ceiling of treatment, mortality, UK

## Abstract

Non-invasive ventilation (NIV) is frequently used as a treatment for acute hypercapnic respiratory failure (AHRF) in hospitalised patients with acute exacerbation of chronic obstructive pulmonary disease (AECOPD). In the UK, many patients with AHRF secondary to AECOPD are treated with ward-based NIV, rather than being treated in critical care. NIV has been increasingly used as an alternative to invasive ventilation and as a ceiling of treatment in patients with a ‘do not intubate’ order. This narrative review describes the evidence base for ward-based NIV in the context of AECOPD and summarises current practice and clinical outcomes in the UK.

## 1. Introduction

Chronic obstructive pulmonary disease (COPD) is a progressive and debilitating respiratory condition characterised by airway inflammation and partially reversible airflow obstruction [1]. COPD is a prevalent disease and represents a leading cause of mortality worldwide. Analyses of data from the landmark ‘Global Burden of Disease Study’ have demonstrated that COPD is the third leading cause of death overall [2,3]. As part of the natural course of the disease, many patients will experience an acute exacerbation of COPD (AECOPD) [4]. The definition of an AECOPD is controversial, with conflict between whether symptom-focussed or event-focussed definitions are optimal [5]. However, a relative degree of consensus exists regarding the 2017 Global Initiative for Chronic Obstructive Lung Disease (GOLD) definition of an AECOPD as “an acute worsening of respiratory symptoms that results in additional therapy.” [6]. A proportion of patients with AECOPD will require hospitalisation for further treatment. Importantly, hospitalised patients with AECOPD experience significant morbidity and around 20% of patients will subsequently develop acute hypercapnic respiratory failure (AHRF) [7]. The pathophysiology of AHRF is complex; alveolar hypoventilation results in impaired removal of carbon dioxide and consequently, hypercapnia. Hypercapnia, in turn, decreases the ratio between bicarbonate ions (HCO3-) and arterial carbon dioxide (PaCO_2_), leading to acidaemia [8].

Crucially, development of AHRF in AECOPD is associated with poorer outcomes, including increased in-hospital mortality and post-discharge mortality [9]. The European COPD audit, conducted at 422 hospitals from 13 European countries, analysed clinical outcomes for 16,016 patients admitted with AECOPD [9]. This study found that the risk of in-hospital mortality was significantly higher for patients with severe acidosis (pH < 7.25) compared to patients without acidosis (pH > 7.35): Odds ratio (95% confidence interval), 2.313 (1.721–3.109). Similarly, in this study, the risk of 90-day mortality was highest in patients with severe acidosis: 90-day mortality was 9.1%, 16.1%, and 28.1% for non-acidotic (pH > 7.35), mildly acidotic (pH 7.25–7.35), and severely acidotic (pH < 7.25) patients, respectively.

In a recent, large, retrospective observational cohort study conducted in the US, Lindenauer et al. [10] used a Medicare database of COPD patients aged ≥65 years to analyse the risk of readmission or post-discharge mortality at one-year in patients who survived to hospital discharge between 2008–2014. The authors stratified the results to compare outcomes in patients receiving mechanical ventilation versus those not. In this study, the readmission rate at one year was 63.5%, 66.0%, and 64.1% among those receiving invasive, noninvasive, and no ventilation, respectively. The mortality rate at one year was 45.7%, 41.8%, and 24.4% among those receiving invasive, noninvasive, and no ventilation, respectively. This study demonstrated that although the risk of mortality following hospitalisation for COPD is highest during the immediate post-discharge period, patients remain at high risk for adverse clinical outcomes for a prolonged period of time. Furthermore, patients treated with mechanical ventilation experience a very high one-year mortality rate.

## 2. Non-Invasive Ventilation in AECOPD

**Figure 1:**
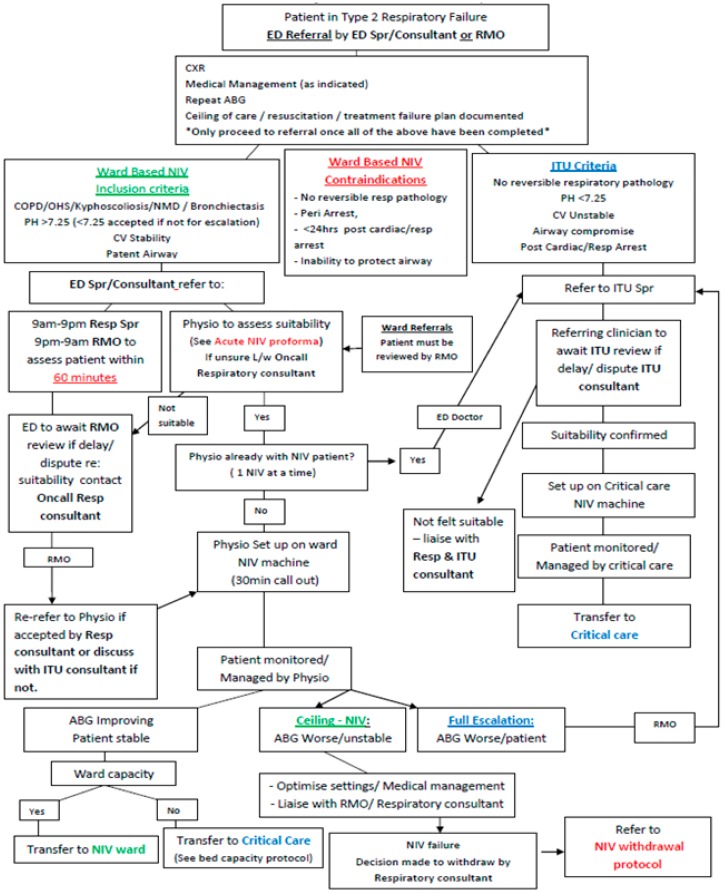
Inpatient acute Non-invasive ventilation (NIV) referral pathway.

Non-invasive ventilation (NIV) is an evidence-based treatment for AHRF in patients with AECOPD. A recent systematic review and meta-analysis found that treatment with NIV, in addition to standard care, for AHRF due to AECOPD was associated with reductions in the rate of mortality and need for intubation of 46% and 65%, respectively [11]. International consensus regarding the evidence in favour of NIV for AHRF in AECOPD is reflected in international guidelines recommending its use in appropriately selected patients [12,13,14]. The British Thoracic Society/Intensive Care Society guideline for the ventilatory management of AHRF in adults recommends utilising NIV in AECOPD patients who remain hypercapnic and acidotic (PCO2 > 6.5 kPa and pH < 7.35) despite ≥60 min of optimal medical management, including controlled oxygen therapy, bronchodilators, steroids, and antibiotics, if clinically indicated [12]. Similarly, the European Respiratory Society/American Thoracic Society guidelines make a strong recommendation for the use of NIV in patients with acute on chronic respiratory acidosis secondary to AECOPD [14]. The same guideline makes a strong recommendation for a trial of NIV in “patients considered to require endotracheal intubation and mechanical ventilation, unless the patient is immediately deteriorating.” A wide range of devices are used in the UK for acute, ward-based NIV; the most common mode is the spontaneous timed (ST) pressure support mode. Most acute hospitals in the UK have ward-based NIV devices, which are also able to deliver an average/intelligent volume assured pressure support (AVAPS/iVAPS) mode, as well as pressure control ventilation (PCV) modes. Figure 1 illustrates the inpatient acute NIV referral pathway for patients presenting to the emergency department with respiratory failure at our institution.

## 3. Development of Ward-Based NIV

Over the past two decades, the use of NIV for respiratory failure in AECOPD has grown rapidly [15]. A particular area of growth in service provision has been seen in ward-based NIV [16]. The landmark YONIV trial (Yorkshire Non-Invasive Ventilation trial) demonstrated that the use of early, ward-based NIV for mild-to-moderate AHRF due to AECOPD results in a significant reduction in need for intubation (15% vs. 27%, *p* = 0.02) and in-hospital mortality (10% vs. 20%, *p* < 0.05) [17]. Since the results of the YONIV trial were published in 2000, numerous other studies have provided evidence in favour of NIV delivery outside of the traditional critical care environment for the treatment of AHRF in AECOPD [18,19,20,21,22,23,24,25,26,27,28,29,30]. Furthermore, ward-based NIV has been shown to be an effective treatment in hospitalised COPD patients with severe AHRF [31]. 

Using prospectively collected data describing COPD patients who underwent NIV for AHRF between 2004 and 2009 at a single centre in the UK, Dave et al. [31] analysed predictors of in-hospital mortality and need for intubation. The authors found that the risk of in-hospital mortality, during a first episode of NIV for AHRF was highest in patients with an admission pH < 7.15 (in-hospital mortality rate = 37.5%). Interestingly, the authors found that in-hospital mortality rates were similar for patients with an admission pH > 7.25 compared to an admission pH 7.16–7.25 during first admission for AHRF (in-hospital mortality rate: 21% vs. 20% respectively). Furthermore, in this study, clinical outcome was not influenced by location of NIV delivery (ward vs. critical care), suggesting that patients with moderate to severe acidosis can be safely managed on a dedicated respiratory ward.

An important consideration in patients with severe AHRF is the requirement for close monitoring of the initial clinical response to ward-based NIV. In a retrospective analysis of COPD patients treated with ward-based NIV, Yalcinsoy et al. [32] found that there were no differences in the rate of intensive care unit admission, in-hospital mortality, or length of hospital stay between patients grouped according to severity of baseline acidosis (7.20 ≤ pH ≤ 7.25 vs. 7.26 ≤ pH ≤ 7.30). These findings suggest that ward-based NIV is safe in patients with moderate to severe AHRF. In a multivariate analysis, the authors identified the following risk factors for NIV failure: A partial arterial oxygen pressure to inspired fractionated oxygen (PaO2/FiO2) ratio < 200 in the emergency room and a delta pH value < 0.30 and a pH value < 7.31 obtained on repeat arterial blood gas analysis within 2-h post-initiation of NIV. The key take-home message from this study is that the improvement in acidosis within the first 2-h of NIV treatment is a stronger predictor of NIV failure than the severity of acidosis on presentation to hospital. It is important to note that the findings of this study are limited by its retrospective observational cohort design; the repeat arterial blood gas analysis will not have been measured following the same length of time after baseline arterial blood gas analysis for all patients. The exact significance of delta pH, as measured in this study, would therefore benefit from further clarification in a prospective cohort. Despite these limitations, the findings from this study suggest that although patients with severe acidosis may be considered for ward-based NIV, these patients should be closely monitored for their response to treatment and, if appropriate, treatment escalated rapidly. 

## 4. Ward-Based NIV as an Alternative to Critical Care Admission

The appeal of ward-based NIV is unsurprising in the context of a universal healthcare system, such as the National Health Service (NHS) in the UK, where the judicious use of resources is a necessity. Ward-based NIV has therefore appeared as a cost-effective alternative to critical care admission [30,33]. The landmark YONIV trial found that NIV use was associated with a reduction in direct treatment costs of £521.41 per patient in 2018 terms [33]. The incremental cost effectiveness ratio (ICER) would be -£1,019.11 per death avoided in 2018 terms, indicating a more effective and less costly strategy. The authors of the YONIV trial report that the cost-savings were predominantly due to reduced utilisation of intensive care units. The cost benefits of ward-based NIV, versus admission to intensive care, are particularly important for low- and middle-income countries with limited access to critical care facilities [34]. Yeung et al. [35] recently published a timely systematic review and Bayesian meta-analysis of randomised controlled trials that evaluated the use of NIV, compared to invasive ventilation, as a weaning strategy in adults mechanically ventilated for at least 24 h. The authors evaluated 25 studies comprising 1609 patients and found that NIV weaning was associated with a lower mortality rate at hospital discharge, shorter duration of invasive ventilation, lower rate of ventilator associated pneumonia, and a shorter length of stay in the intensive care unit. The benefits of NIV weaning were most notable in patients with COPD, suggesting that critical care resource utilisation may be reduced by using an NIV weaning strategy in this patient cohort.

Results from a review of international surveys demonstrate that NIV use for AECOPD is increasing worldwide [36]. The authors suggest that this is due to both an increase in the evidence-base in favour of NIV in AECOPD, and an increase in clinician experience of administering NIV. In line with previous studies, the authors suggest that high risk patients should be monitored closely, and treatment escalation should be considered, including timely critical care admission and invasive mechanical ventilation, if patients do not adequately respond to NIV treatment. 

## 5. Ward-Based NIV in Patients with a ‘Do Not Intubate’ Order

In a recent systematic review and meta-analysis of observational cohort studies, Wilson et al. [37] investigated outcomes following acute NIV in patients with ‘do not intubate’ orders or ‘comfort measures only’ orders. The authors found that a significant proportion of patients with a ‘do not intubate’ order survived to hospital discharge and one-year following acute NIV. Pooled survival to hospital discharge in COPD patients with a ‘do not intubate’ order, treated with acute NIV was 68% (95% confidence interval, 53%–81%). This study also found that survival to hospital discharge was no different between patients who received acute NIV on ‘well-equipped hospital wards’ versus the critical care environment (pooled survival to hospital discharge (95% confidence interval): 65% (59%–71%) vs. 58% (46%–69%), *p* = 0.32). Furthermore, although data was limited, the results of this study suggest that there was no decrease in quality-of-life in survivors following acute NIV. The findings from this study provide additional data supporting the use of NIV for AECOPD outside of the critical care setting, in patients with NIV as their ceiling of care. This study also raises an interesting debate regarding what constitutes a ‘well-equipped hospital ward’. Based on current UK practice, a hospital ward well-equipped for acute NIV would usually include a dedicated clinical area with the following characteristics: (a) A multidisciplinary healthcare team who have undergone structured competency training (most commonly led by physiotherapists or nurses) for NIV delivery; (b) a written standard operating procedure/guideline on application and weaning of NIV; (c) a formal or informal sliding scale of collective patient acuity with the ability to increase or decrease nurse-patient staffing ratios based on acuity on a daily basis; and (d) daily reviews of patients by a respiratory specialist with 24-h access to emergency medical support. 

Interestingly, in our institution, we have observed an increase over the past decade in the number of COPD patients treated with acute, ward-based NIV who have NIV documented as their ‘ceiling of treatment’ (unpublished data). This may, in part, have resulted from an increased emphasis over recent years on the importance of discussing and documenting treatment limitations with patients and their families before an acute deterioration in their health. An example of this in the UK would be the development of the Recommended Summary Plan for Emergency Care and Treatment (ReSPECT) process (https://www.respectprocess.org.uk/). It is feasible that some clinicians may feel reluctant to admit patients who have treatment limitations for acute NIV, which may be due to a sense of prognostic pessimism and a perception that the use of NIV in this patient cohort may simply prolong the dying process (37). However, this is not clearly reflected in the data from our institution. The increasing proportion of COPD patients with treatment limitations necessitates a greater understanding of the role of acute NIV in this patient cohort to optimise clinical outcomes. A palliative approach may be appropriate in selected patients with treatment limitations in whom NIV fails, but this should be determined on an individual patient basis, considering an assessment of benefits versus burdens of treatments and patient preferences. Further research is required to clarify the role of acute, ward-based NIV in COPD patients with treatment limitations, including ‘do not intubate’ orders and ‘ward-based care only’ decisions.

## 6. ‘Real life’ Outcomes following NIV in the UK

Concerns have grown in the UK regarding a higher mortality rate following acute NIV than would be expected based on the original randomised controlled trials. Reasons for this discrepancy are likely to be multifactorial, however, a major factor is likely to be the difference in the population seen in the universal healthcare provision within the UK NHS, where ‘real life’ studies and audits have shown that around two-thirds of the recipients of acute NIV do so as the ceiling of care. In 2009, the National COPD resources and outcomes project (NCROP) conducted an audit of NIV practice in the UK [7], which identified several areas of NIV service provision in the UK which were of concern, including patients receiving NIV inappropriately and, in some cases, eligible patients failing to receive NIV. Building upon this data, between 2010 and 2013, the British Thoracic Society conducted annual, national adult NIV audits to monitor mortality rates and identify any deficiencies in country-wide practice [38]. These national audits demonstrated a climbing mortality rate and wide variation in practice. Consequently, a National Confidential Enquiry into Patient Outcomes and Death (NCEPOD) review of adult NIV was launched to try and identify causes for the climbing mortality rate in the UK [39]. 

NCEPOD brought together an expert, multidisciplinary group of healthcare professionals to try and identify any avoidable causes of increased mortality and deficiencies in the care of patients receiving acute NIV. The project administered questionnaires to both individual clinicians and organisations involved in the delivery of acute NIV. In addition, NCEPOD requested case notes for 353 individual patients to facilitate peer review and to conduct a detailed evaluation of individual cases of acute NIV. Despite its limitations, the NCEPOD provided a wealth of data regarding the quality of acute NIV delivered in UK hospitals. Importantly, NCEPOD identified numerous shortcomings in the quality of care delivered to these patients. In patients with COPD treated with acute NIV, the in-hospital mortality was found to be 25.1%. The NCEPOD report makes a total of 21 recommendations to improve the quality of care provided to patients receiving acute NIV. Recommendations include a requirement for an acute NIV clinical lead at each hospital and an operational policy, which includes definitions of: Appropriate clinical areas where acute NIV can be provided, minimum safe level of staff competencies and staff to patient ratios (minimum of one nurse to two acute NIV patients), treatment escalation plans (defined prior to NIV treatment), step-down care procedures, standardised documentation, minimum frequency of clinical review, and seniority of reviewing clinicians [39]. NCEPOD also highlight that an episode of acute NIV should act as a prompt to consider palliative care referral for symptom control and/or end-of-life care and that all patients surviving an episode of acute NIV should have future treatment plans discussed and documented. Following the NCEPOD report, the British Thoracic Society released a series of ‘Quality Statements’ to help guide improvement in delivery of acute NIV, as summarised in Table 1 [40]. The next British Thoracic Society adult NIV audit is planned for early 2019 and will provide important data on whether recommendations from the NCEPOD report have affected practice in the UK.

## 7. Conclusions

Hospitalised patients with AECOPD experience significant morbidity and mortality. Ward-based NIV is an evidence-based treatment in appropriately selected COPD patients with refractory AHRF. International guidelines support the use of NIV in AHRF due to AECOPD and there has been a worldwide increase in the utilisation of ward-based NIV in these patients. Current evidence suggests that patients with severe AHRF secondary to AECOPD can be considered for ward-based NIV, where a dedicated area with appropriately trained staff is available and patients can be monitored closely for their response to treatment. Recipients of ward-based NIV should have a clear, documented escalation plan, including timely review by appropriately trained clinicians. Quality improvement in the delivery of acute NIV is vital to improve clinical outcomes in the UK.

## Figures and Tables

**Table 1:** British Thoracic Society Quality Statements for acute NIV in adults.

1	Acute NIV should be offered to all patients who meet evidence-based criteria. Hospitals must ensure there is adequate capacity to provide NIV to all eligible patients.
2	All staff who prescribe, initiate, or make changes to acute NIV treatment should have evidence of training and maintenance of competencies appropriate for their role.
3	Acute NIV should only be carried out in specified clinical areas designated for the delivery of acute NIV.
4	Patients who meet evidence-based criteria for acute NIV should start NIV within 60 min of the blood gas result associated with the clinical decision to provide NIV and within 120 min of hospital arrival for patients who present acutely.
5	All patients should have a documented escalation plan before starting treatment with acute NIV. Clinical progress should be reviewed by a healthcare professional with appropriate training and competence within 4 h and by a consultant with training and competence in acute NIV within 14 h of starting acute NIV.
6	All patients treated with acute NIV should have blood gas analysis performed within 2 h of starting acute NIV; failure of these blood gas measurements to improve should trigger specialist healthcare professional review within 30 min.
